# The Treatment of Surgical Tuberculous Affections with Tuberculin (T.R.)

**Published:** 1908-11-28

**Authors:** 


### SURGERY.

### THE TREATMENT OF SURGICAL TUBERCULOUS AFFECTIONS WITH TUBERCULIN (T.R.)

THE treatment of tuberculosis attacking other organs than the lungs has until quite recent times consisted of surgical interference only. But it must be confessed that the results of such treatment are on the whole exceedingly discouraging to the thinking surgeon. To take the case of a tuberculous hip or knee which has been treated by all the known forms of palliative treatment without improvement, and has finally been excised, the result must be regarded as highly unsatisfactory, to say the least of it. For even if the disease is entirely eradicated, which unhappily is not always the case, the patient is permanently maimed and crippled in a more or less serious degree. And even in less unfavourable cases surgical interference can only be partially successful, since it aims merely at the removal of the focus and does nothing towards helping the patient to overcome the infection. In other words such treatment does not rest on a scientific basis. Any method, therefore, which holds out a prospect of permanently curing the disease without disfiguring or disabling the patient, should be regarded with gratitude by the profession at large.

Much work has recently been done on the results obtained by the administration of tuberculin (T.R.). Sufficient time has not yet elapsed to enable us to estimate the ultimate value of this method as a therapeutic agent, but the results which have been so far attained are encouraging.

Any localised tuberculous lesion must be regarded as a factory of tuberculo-toxin which is readily disseminated throughout the system. Such dissemination is brought about by exercise or movement of the affected part; and if an excessive amount is liberated in this way, it is injurious to the individual, as indicated by a rise in his temperature chart, a fall in his opsonic index, and a feeling of malaise. This can be shown by observing the effect of unregulated exercise upon a phthisical patient or, for instance, by inducing passive movements in a tuberculous knee. This process is known as auto-inoculation. But in lesser amounts such an auto-inoculation may even do the patient good by increasing his resistance to the infection. It has, therefore, been argued that in cases in which surgery has been apparently successful, as in excision of tuberculous glands of the neck, a sufficient amount of tuberculo-toxin was massaged into the lymphatic system by the manipulations of the operation to complete the patient's cure and to render him immune to reinfection.

It might then be argued that all one has to do in such a case is submit the patient to massage or graduated exercises, so that he can supply his own vaccine to promote his cure.

This would be an ideal system if it were only possible; but unfortunately it is not feasible, because it is difficult to calculate the degree of auto-inoculation which will be produced. Instead of this, however, we can administer tuberculin in known doses which produces a definite observable effect. In view of the fact that an overdose of tuberculin may produce injurious results, it is well to start with a very small dose, say $\frac{1}{1000000}$ of a milligramme, and this can be gradually increased until the correct amount is being given. It is not a matter of vital importance whether the tuberculin is given by the mouth or is injected subcutaneously. The first indication that the dose is having any effect, will be given by the temperature chart. The daily oscillations which are always observable in the chart of a tuberculous patient will become gradually less and the mean temperature will come down to near the normal line. Some cases of surgical tuberculosis are apyrexial, that is to say that although the temperature chart oscillates the highest point is never above normal. In this case we have to rely simply on the levelling of the chart, the variation between the morning and evening temperature becoming eventually negligible. In addition to this a patient who is taking a proper dose of tuberculin will admit to feeling a distinct improvement in his general condition. It follows that the dose given can be regulated by an accurate observation of the clinical course of the case. Important information can also be obtained by taking the tuberculo-opsonic index. But a word of caution is needed upon this point. If it is to be of any value it must not be taken casually. It is not sufficient to take the index, for instance, on one occasion, and to deduce that the patient necessarily has tuberculosis because that index is low. It is necessary to know what the patient's temperature was at the moment the blood was taken. For we find that the chart of the index varies inversely with that of the temperature. Thus the index of a tuberculous patient may be above normal if the blood was taken from him at a moment when his temperature was subnormal. A series of observations must be taken, therefore, and if it is found that in each case the index varies inversely with the temperature chart it may be concluded that the patient is suffering from tuberculosis.

Let us take a hypothetical case. A patient with supposed tuberculosis of the testis is given a small dose of tuberculin, say, $\frac{1}{50000}$ of a milligramme. No clinical effect is produced. A second dose is given of $\frac{1}{20000}$ of a milligramme. The chart at once becomes level, the index is found to vary inversely as the chart, and the patient feels better. That is a case in which tuberculin is having good effect. As soon as the temperature chart begins to oscillate again, which it will do as soon as the effect of the tuberculin begins to wear off, a further dose is given; and if this method is continued the local lesion undergoes enormous improvement or clears up completely.

The lesions which have so far been found to react most readily are tuberculous peritonitis, cystitis, and lymphadenitis. But one thing must be remembered, especially in the latter case, that tuberculin is unable to remove caseous material; and if this has already been formed the administration of tuberculin must be combined with surgical interference.

